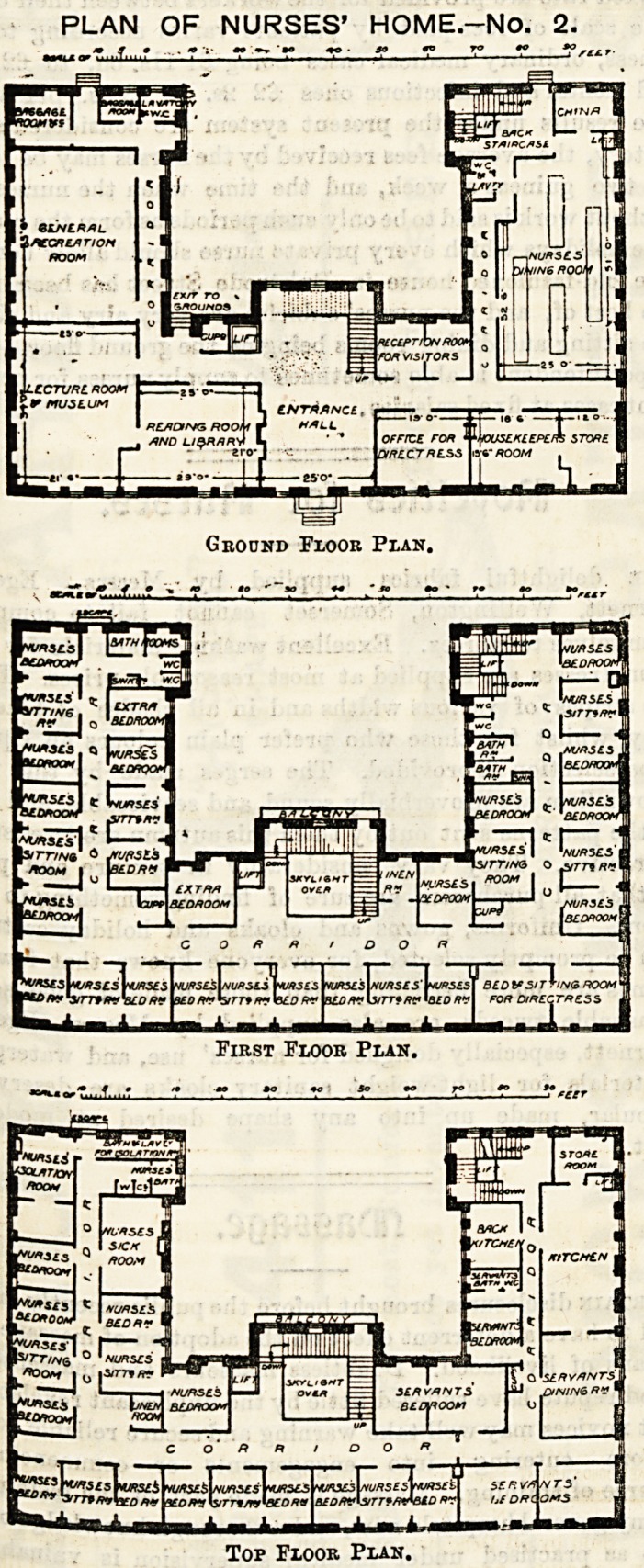# The Hospital Nursing Supplement

**Published:** 1894-10-06

**Authors:** 


### "The Hospital" Nursing Mirror.

Being the Extra Nursing Supplement of "The Hospital" Newspaper.

[Contributions for this Supplement should be addressed to the Editor, The Hospital, 428, Strand, London, W.C., and should have the word "Nursing" plainly written in left-hand top corner of the envelope.]

### News from the Nursing World.

### "THANK GOD THERE ARE NURSES!"

The boy had small-pox—a very bad attack—and it was thought only right that his mother, a poor widow, should be informed that her son's condition was very serious. She had been vaccinated herself, although she had failed to observe the same precaution with regard to him. She did not fear infection, considering "if people *was* to have things they'd catch 'em fast enough." She presented herself at the ward door soon after receiving the doctor's communication respecting Tommie. "You will find him looking very bad," she was warned, as she approached the bed. She looked at the swollen disfigured face, the closed eyelids, the wandering hands plucking at the bedclothes; and then she glanced at the white card over the bed, "Thomas Simpkins" was inscribed upon it. She looked again at the face, and with unconcealed loathing exclaimed, "How dare you play me such a trick, nurse! It's my boy's name right enough, but you ain't got no business to shove it up over such an object as this! Call it a boy, indeed; why, I never see such a 'orrid sight in all my life; it's more like a"——but "it" gave a little sigh, and muttered, "Mother, mother; it's my mother come at last, and I'm too blind to see her!" But the woman shrank back from the poor little figure; "Thank God as there's nurses!" she muttered; "for, may the Lord forgive me, but I could never bring myself to touch him now!"

### DR. JAMES ANDERSON.

Special interest attaches to a little volume entitled "Medical Nursing," just published by Mr. Lewis, of 136, Gower Street. It is compiled from the original notes made by the late Dr. James Anderson for his lectures to the nursing staff at the London Hospital. The introductory biographical notice is taken from the last presidential address delivered by the late Sir Andrew Clark at the Royal College of Physicians, and a more suitable opening chapter could not possibly have been chosen. Dr. Anderson's notes have been admirably edited by Miss Lamport, formerly a sister at the hospital where the memory of both physicians is affectionately and reverently cherished.

### MATERNITY WORK.

In November, 1892, the maternity work at St. John's House, Battersea, was taken over by the lady doctors who so successfully carry on the Clapham Maternity Hospital in Jeffries Road, Clapham. By them St. John's House was closed as an in-patient institution, it being felt that this was no longer needed, and it became solely an out-patient department under the charge of two resident qualified medical women. At first only one nurse was employed, it being thought wise to make a very modest beginning as the St. John's Association had given up the management on account of its expense. After nearly two years' experience the maternity institution is found to work exceedingly well on the new lines, and Albert House, Albert Road, has recently been taken, and a matron added to the existing staff. Miss Wainwright was trained at St. Bartholomew's Hospital, holds the L.O.S. diploma, and was for several years matron of the Jenny Lind Hospital, Norwich. She now supervises the nursing connected with the Battersea District Maternity, and trains suitable candidates in district maternity nursing. Last year 508 cases were attended by the doctors and pupil midwives, and it is evident that the training at Battersea must be most valuable for those who propose to take up district nursing after they have gone through an adequate course of general hospital training. Such complete equipment for the responsibilities of district nursing cannot be too often insisted upon, and we have pleasure in announcing the new movement at Battersea, to which we wish all success so long as it steers clear of the fallacy that district nursing and maternity work can be mastered in three months, by novices in nursing.

### A VALUABLE CITIZEN.

The death is announced of Mrs. Melville Green, whose name is associated with so many useful undertakings at Worthing. She was elected a lady Guardian of East Preston Union some six years ago, and her services were publicly acknowledged in Sir Henry Fletcher's speech on the new Local Government Board Act. Mrs. Green took a great interest in Home Nursing, and was a member of the local Technical Instruction Committee. She had a great deal to do with the District Nursing Association, and her labours during the epidemic of typhoid will long be remembered by her fellow workers on the Hospital Committee at that time. Mrs. Green will be greatly missed by those to whom she had endeared herself in her busy and useful life.

### A NURSE'S DIAGNOSIS.

The report of the inquest on a pauper who cut his throat in Plymouth Workhouse Infirmary the other day calls attention to the fact that the nurse did not summon help after the occurrence of the tragedy because she considered the case hopeless. A discussion arose at a subsequent meeting of the Board of Guardians as to the efficiency of the nurse, on which various opinions prevailed. A Lady Guardian had the courage to say that she "considered the nursing unsatisfactory," and in that opinion other people will probably agree.

### NURSING AT CHIPPENHAM.

The Chippenham Board of Guardians have decided to let their medical officer get them a properly certificated nurse to fill the vacant post in the infirmary. At a recent meeting the following resolution was passed, "That the matron be informed that any complaint against the hospital nurse must be brought directly to the notice of the Board or through the Visiting Committee, and that the nurse be also informed that any complaint or recommendation she may have to make must be made in the same manner, and that both the matron and nurse be prohibited from communicating any complaints to any person other than a Guardian."

### PLEASANT INSTRUCTION.

Intelligent children object to the story book written for their instruction, and even adults like to have a bitter dose of medicine pleasantly flavoured. The drug does its work when agreeably disguised, and the tale is eagerly devoured if its lesson is judiciously dispensed. Miss Isabel Dodgson must have had these facts in her mind when she decided to illustrate the talks on practical nursing in cottages which she has been giving in Oxfordshire during the last two years. She is now commencing her third winter campaign, and her modest amount of personal luggage is supplemented by some mysterious-looking cases which tend greatly to popularise her course of "lectures." With a model bedstead she shows how a patient's sheets can be changed, and then she proceeds, by means of some excellent magic-lantern slides, prepared by herself, to still further amplify the lesson. The worst possible arrangements of the cottage bed-room are shown just as plainly as the best methods of using the means at command. All modes of ventilation, with and without draughts, are admirably depicted on cleverly-designed slides. A home-made bed-rest, cradle, arrangements of pillows, temporary screens, and numerous personal and structural points are also shown by Miss Dodgson, who certainly has learned to convey instruction without unduly emphasising "the moral," wisely implied rather than expressed.

### SOLDIERS' WIVES.

Soldiers' wives appear to be fortunate in the Garrison Women's Hospital they have at Woolwich. There has not been a single death from puerperal fever for nine years, and Miss Dryland, the matron, with her excellent staff of trained nurses, may well be proud of the contented-looking patients and their fine healthy babies.

### AN INDEPENDENT INSTITUTION.

The second annual report of the Peter Brough District Nursing Association, which is affiliated with the Queen's Jubilee Institute, is most satisfactory. It enjoys the enviable distinction of being the only institution of the kind in Scotland which does not receive pecuniary assistance from the public. The entire support of the Home is provided for by the generous bequest of the late Mr. Peter Brough of Paisley. The report shows that grateful patients have given donations to the amount of £8 1s., this fund being devoted to the purchase of a bath chair and to giving country drives to convalescents, welcome boons to those recovering from tedious illness. The Convalescent Home at West Kilbride has received thirty-five of the patients, and two have stayed at the Helensburgh Children's Convalescent Home. The staff consists of the Superintendent, Miss Watson; and five Queen's Nurses, Cowper, Colvin, Farrell, Bayne, and Davidson.

### QUEEN'S NURSE AT FORFAR.

The second annual meeting of the Forfar District Nursing Association was held in the Town Hall, and presided over by the Earl of Strathmore. The association is affiliated with the Queen's Jubilee Institute, and the work of Miss Foulkes, the nurse, appears to be highly valued by the sick poor of the neighbourhood. Visits were paid to 146 cases in the course of the year, some of them very critical ones, demanding most skilful nursing. Great interest in the association was manifested by the large assembly of ladies and gentlemen who were present at the meeting; in fact, district nursing seems to be thoroughly well established and cordially supported at Forfar.

### DIVERTING THE PATIENTS.

Many are the methods used to attract or to divert the juvenile patients who come to be interviewed in the doctor's consulting room, and it is wonderful to see how successfully wise men divine the tastes of these little ones. A special instance comes under our notice, for the aged Dr. Heinrich Hoffman, who died suddenly at Frankfort on September 20th, wrote, amongst others, the ever-popular "Struwwelpeter," the sketches being dashed off in pencil for the amusement of children. He was a specialist in mental diseases, and wrote many books and much poetry; but we have named the best-known one, which, to the surprise of the author, ran into a hundred and forty editions, and has been translated into most languages.

### THE McLEAN HOSPITAL.

The new buildings of the McLean Hospital (for the insane) are now nearly completed, and are situated at Waverley, a short distance from Boston, in a park of 170 acres. One of the special features is a gymnasium for women patients, to be under the charge of the resident instructor in physical training. The nurses will also receive from this lady systematic instruction. The male patients will have a gymnasium for themselves, and the new hospital bids fair to be structurally one of the most complete institutions in existence for the treatment and care of the mentally affected.

### SHORT ITEMS.

The Loughton and District Nursing Association requires fresh subscribers to enable the good work to be carried on satisfactorily.—The Hampstead Board have appointed a permanent nursing committee, which includes two ladies, for their workhouse infirmary.—The resignation of Miss A. J. Lloyd, who has held the post of matron very successfully at the hospital at Stratford-on-Avon, has been received with much regret by the committee.—A rummage sale and tea recently held in Osbournby, under the patronage of Lady Whichcote, passed off successfully. It was held to raise funds for the institution of a trained district nurse for several parishes.—*Nursing Notes* for October contains an excellent article on women as guardians of the poor, and many other matters of interest to nurses.—A Convalescent Home for Soldiers has been established in the neighbourhood of Dresden by the Saxon Ministry of War.—The Bridgend and Cowbridge Guardians have decided "to effect a reform in the nursing of the sick poor at the workhouse" by appointing a certificated trained nurse.—There is a scheme on foot to secure trained nurses for the sick in the parishes of White Waltham, Boyn Hill, and Littlewide.—A committee has been formed to organise an institute at Sandown to provide a competent district nurse for the poor in the parishes of Christ Church and St. John's.—Mr. Starley and Messrs. Chiley have presented two bicycles to the Coventry Nursing Institution for the use of the district nurses.

### Our Work.

Reflections in the "Nursing Mirror."

"The Hospital" year begins with this number, which is dated October 6th, and by means of "The Mirror" nurses will be kept in the future, as they have been in the past, in close touch with each other.

Fresh features will be added to the Nursing Supplement and new friends will no doubt join the huge circle of old ones.

Questions and Answers.

It really seems sometimes as if "Our Work" consisted chiefly in answering questions, so many communications reach us commencing with a query. Some of these can be answered immediately, but others require quite a serious amount of consideration and research. All questions, however, do get replied to eventually, whether easy or difficult, as long as the writer gives full name and address. Anonymous correspondents are deservedly disliked, although in some cases the omission of a proper signature may be an act of carelessness only. From "where to buy a thermometer" to "the best place to winter abroad" there is a wide range of questions, and really it seems to be pretty well covered in the course of the year, but we are quite willing to go on cheerfully replying to all questions.

A Fund of Information.

When the Editor of The Hospital first conceived the idea of devoting a supplement to nursing matters, it was not difficult to foresee that the day would come when nurses would turn instinctively to its pages for help and information of all sorts. The disabled or sick nurse gets advised and assisted; the vigorous give and receive information through its columns; the nurse abroad reads the home "appointments," and so she is enabled to trace her old friends progress in the career with which they have both identified themselves.

Our Christmas Competitions.

Our Christmas Competitions have become familiar to all, and we reckon on the parcels increasing annually in size and in number. This season, therefore,

A New Development of the needlework competition is resolved upon, and our readers should take note thereof. It is found that many people would be glad to contribute a garment, but they fancy they lack either time or skill for becoming actual "competitors." In such cases a label with "Not for competition" may be attached to each offering.

Time without Money is possessed by some Hospital readers who say they would gladly give the former if they could be provided with materials by those who have

Money but no Spare Time, and in this way a combination might evolve many good outfits. Perhaps no one who is not a nurse can quite realize the clothes a patient wears on admission to hospital. The minority are clad in decent and cleanly garb, but even these oft-washed garments are very thin, and the under-vest, if existent, is a mere cobweb! For poor "medical cases" something warm is as essential as the tonic which the doctor insists must be taken "for another month at least." But the majority of patients have garments unutterably deficient in quantity and quality. To send such out of the wards without any addition to their scanty wardrobes needs a harder heart than a nurse's.

Something Must be Done.

The supply in the clothes' cupboard is generally low, and "useful things" are sorely needed in the winter. "Just before Christmas" is a time of great poverty in many a home, and when the earnings are precarious the meals are apt to be scanty and there is no surplus for clothes or for boots! "Their feet are always on my mind in the winter," remarked a ward Sister. "I'm sometimes afraid chance visitors will catch me appraising their shoes and stockings. I find my eyes wandering downwards whilst I mentally wonder how soon the present owner will give them away, and if they'll come into my ward!"

Stockings and Socks.

Knitting is so seasonable and can be done at moments when other work would be impossible, and just fancy the noble results of every reader of The Hospital making one pair of stockings or two pairs of socks annually! That would be very little for each individual to accomplish, but what a splendid total there would be!

More!

As regards warm garments of all sorts we can but reiterate our constant appeal for more, and yet more. The gifts that come are so warm and pretty and serviceable that they give immense pleasure, their value being increased by knowledge of the kindness which provides them, but we want the number multiplied exceedingly.

Unaccustomed Recipients.

There is a difficulty in choosing the Christmas presents in prosperous households. "They have so many things. I don't know what to give them," is a familiar plaint; but with our sick poor there is no such puzzle to solve. They have so little of anything, that everthing is welcome, and it may be taken for granted that there are very few sick persons occupying hospital beds on Christmas Day who are not in sore need of a garment of some sort to don on the day of departure.

Women's Work.

Nature certainly did not design every unmarried woman for a nurse, and our articles under the heading of "Woman's Work" have shown to our readers some of the careers which can now be followed with more or less success by girls and women. Each day some fresh branch of industry seems to be taken up, and of the best of these we propose from time to time to write.

Our Examinations.

These have continued to enjoy much popularity, and letters have been received from many distant places during the year acknowledging gratefully the helpfulness of the questions.

### "The Hospital" Convalescent Fund.

Earlier in the year we promised to give an account of the work accomplished during the summer through the generous contributors to The Hospital Convalescent Fund. Every application for assistance has proved satisfactory, and we have had the great pleasure of providing the means for a month's rest and change to those who would have been quite unable to resume their occupation without it. It has been a delightful task opening the letters full of appreciation and gratitude. Our first applicant this summer, Nurse M., had broken down from overwork, and after a serious illness had no means of providing the necessary change of air. Nurse M., besides making a provision for the time when working days should be over, contributed to the maintenance of a younger sister; therefore in time of sickness she was a fit and grateful case for help from the Convalescent Fund. Nurse M. returned from a visit to the country restored and is at work again. In the case of Nurse P., our next applicant, recovery was not so speedy. She had been ill for a long period, and had exhausted all her savings. We learnt, too, when visiting her, that her wardrobe sadly needed replenishing. This we scrupled to do through the Convalescent Fund, so the kind member of our own staff who visited her, started an independent collection, which not only secured Nurse P. a neat cloak, bonnet, and a pair of gloves, but has left us enough in hand to help again if necessity arises. Nurse P. spent a month at the seaside, and was certainly more fit for work at the end of it than when she arrived.

We were fortunate in securing accommodation at the same comfortable Home which received Nurse P. for Nurse G. The change proved greatly beneficial to her. After a long and serious illness she found herself at the end of her resources. Her stay of one month, which we arranged for through the Fund, was prolonged to six weeks, as the kind sister-in-charge suggested that she should remain in exchange for a little assistance which she could render at the Home. Shortly after her arrival she wrote: "I certainly could not wish for a more quiet and refreshing spot, and trust and believe that I shall return to London well." Nurse G. was still so far off convalescence as to require a milk diet for some time after her arrival at the Home.

Nurse B. is still enjoying the benefit of the seaside. She has been ailing for some time, and greatly in need of change and comfort. In the hope that her great wish to resume work might be furthered, we gained admission for her into the kind home, which we hope will be able to receive all our ailing nurses within reasonable travelling distance from it. Nurse B.'s own words are the best testimony to her satisfaction. She writes: "Altogether it is a delightful change—I do fully appreciate and enjoy it." This we feel is the return for their kindness to those less fortunate, which will please our subscribers most, and we hope one and all will take to themselves a due share of the pleasure the power to help thus entails. We are always glad to receive donations, but shall not make a special appeal until necessary, as we have full confidence, born of experience, that we shall never ask of our readers in vain. We, for our own part, spare no trouble to ensure that what is entrusted to us is laid out in the best possible manner. We are now in correspondence with several applicants for "a change to the sea," and if our help is needed to a sufficiently large extent we shall publish a further record in the spring.

### Scattered Nurses.

An excellent article on the nursing of yellow fever which recently appeared in The Hospital graphically indicated the nature of a nurse's duties in that terrible disease. It is exceedingly interesting to receive the personal experiences of observant nurses in epidemics, and those on yellow fever and the plague at Hong Kong were reliable and instructive. From Labrador comes news of admirable work, done by trained English nurses, for those sorely needing it. In India nurses have the drawbacks of climate and isolation added on to their professional duties. In the colonies native help in domestic matters is so uncertain in quantity and quality that the work of the nurse seems unlimited.

It is well to think of the surroundings of some of our sister nurses, and those whose career is entirely English can hardly realise how much harder life may be in some of the lands from which The Hospital Nursing Mirror has reflected scenes during the past twelve months. Articles have been inserted penned by nurses in England, Scotland, Ireland, Wales, France, Germany, Holland, Spain, Switzerland, Canada, the Labrador, the United States, Mexico, Brazil, Japan, China, South Africa, Victoria, New South Wales, South Sea Islands, West Indies, Ceylon, and India all showing evidence of good and faithful work.

In England itself there are also nurses who live isolated lives bravely and patiently, unconscious of their heroism. The district nurse who works single-handed stands very much alone, although she is not solitary, for her duties bring her into close contact with her fellow-creatures. The nurses in infectious hospitals are also much cut off from their fellows. If the busy hospital nurse, or the prosperous private one, would spare an occasional thought to the colonial, district, and fever nurses, she would find many opportunities of keeping in touch with some of them.

"To whom shall I send it?" occasionally write correspondents of The Hospital, explaining that some weekly or monthly journal, which is taken regularly, would be at the service of a fellow worker if a suitable recipient were found.

Of course a recipient is always forthcoming, and the link thus cemented becomes a strong one, and the periodical regularly dispatched is eagerly anticipated, as a token of kindly remembrance, as well as news from the busy outer world.

### District Nursing.

In few departments of nursing has such rapid progress been recently made as in this. Some years ago "Perhaps she will do for district work" was commonly said with regard to well-meaning but incompetent probationers, whom no amount of training could convert into efficient ward nurses. It was supposed that rudimentary instruction could evolve a nurse "quite good enough" for this out-door work. Some of those individuals are still to be found trudging around scattered districts and doing valuable service in their own way; but they are apt to forget that much of their practical usefulness was painfully acquired by means of a blundering experience, and their present knowledge tempts them to underrate the strict training which secures for the work efficient women nowadays.

### Queen Victoria Jubilee Institute.

There is always a demand for qualified district nurses, and all particulars as to the rules of the Queen's Jubilee Institute can be obtained from Miss Peter at St. Katharine's Hospital, Regent's Park, London. Many provincial nursing associations are now affiliated with the Institute. This secures to them a supply of "Queen's nurses" and systematic supervision of the work done by them, the "inspectors" being themselves trained nurses.

### The Central Training Home of the Queen's Nurses is in Bloomsbury Square, London, and there women who have already secured adequate hospital training are practically instructed in nursing the sick-poor in their own homes.

### Cottage Nurses cannot properly be classed with "district nurses," as they are not necessarily trained in anything besides monthly nursing, although they pick up some useful general knowledge in their course of three or six months' maternity work.

### The Name Without the Knowledge.

The inconsistency is obvious of letting loose on the rural community numbers of women whose knowledge is perfunctory, whilst they are outwardly indistinguishable (by ignorant patients) from the real district nurse. However, they certainly aspire to usurp her place, having already taken her title, and in some cases her uniform.

### The Queen's Example.

Her Most Gracious Majesty has set a high standard before her subjects in this as in many other instances, and it would be well for all who are embarking on new schemes for the benefit of the sick poor, to make a careful study of the plan formulated by persons of large experience, and approved and supported by the Queen. The fundamental principle of the Council of the Q.V.J.I. is a determination to secure for the sick-poor attention and skill in no way inferior to that provided for the richest in the land. Fortunately for the nation, her Majesty's philanthropy has never allowed her to consider "anything" good enough for the poor, and her example in securing skilled nursing for them may well be followed by those who have less knowledge of the subject than the Queen herself possesses.

For Nursing in Metropolitan Fever Hospitals, &c., see p. v.

### Nursing in Metropolitan Fever Hospitals.

Regulations.

The manual of regulations for the nursing staff at the fever hospitals of the Metropolitan Asylums Board has been recently revised, and is now a fairly complete code. A copy of the little pamphlet is to be given to each nurse and assistant nurse when she enters the service of the Board.

Salaries.

Charge nurses are appointed at annual salaries of £36, rising £1 per annum to £40, and assistant nurses (Class I.) at £24, rising £1 per annum to £28, whilst assistant nurses (Class II.) begin at £20, rising £1 per annum to £24. These increases are made on the recommendation of the medical officer and matron. Board, lodging, uniform, and washing are all provided, and the nursing staff rank as a separate and superior class to the other members of the female staff.

Time Off.

Each nurse has twelve hours leave in the course of each week and twenty-four hours once a month. Charge nurses get a month's annual holiday and the assistant nurses each have three weeks.

Qualifications of Charge Nurses.

Charge nurses must be at least 25 years of age, and must hold satisfactory certificates for three years' training in a general hospital with a recognised training school, or from "a poor law infirmary in which systematic instruction is given and tested by subsequent examination by an independent authority."

Assistant Nurses in Class I. must be 23 years of age, with one year's general training. They will be required to serve two years in the metropolitan hospital before being made charge nurses. For Class II. applicants need have no previous training, they must be 22 years old, and will serve at least one year before being eligible to become assistant nurse, Class I.

Promotion takes place in all cases on the recommendation of the medical superintendent and matron, whose directions, as well as those of the night superintendent, the nurses are expected to obey.

Information as to the careful disinfection of clothes and person insisted on before a nurse goes out of the hospital are also given in this manual, and surely no nurse, after reading it, will ever be able to assert that she "did not know what was expected of her!"

Certainly the revised rules show that it is the intention of the Metropolitan Asylums Board to govern their nursing staff with justice and consideration.

### Army Sisters.

The present regulations as to the amount of training which nurses must undergo before being accepted as "Nursing Sisters" ought to secure excellent attendants for our soldiers at home and abroad. Trained nurses are also being provided in some places for their wives and children.

### Mental Nurses.

There seems a prospect that the standard of asylum attendants will be much higher in the future than in the past, and doubtless uniformity of instruction and examinations will do much to promote this. Thoroughly trained and certificated mental nurses command good fees, but the work is of a most responsible and anxious character.

### Nursing in Workhouses.

This subject has justly engrossed a great deal of attention of late, and although some boards of guardians are perversely setting their faces against every proposed improvement, others are pursuing a wiser policy. That many grievous evils exist, that the sick, the infirm, and the imbeciles are still without efficient nursing in many unions, is too widely known to be any longer questioned.

The Workhouse Infirmary Nursing Association has been struggling for years to replace pauper helpers by trained nurses in sick wards, and their efforts have been appreciated in numerous institutions. Now that the tide of public feeling has set so strongly in their favour the demand on their resources is an ever-increasing one. When guardians are found anxious to improve the condition of their infirmaries by engaging qualified nurses, it is grievous to hear that want of funds seriously curtails the work of the Association.

Inadequate Support.

It appears as if the patient efforts of a little band of women were deemed too insignificant to arrest the tide of charity which flows by the doors of the modest office at 6, Adam Street, Strand, and yet few objects are more deserving of support.

Untrained Matrons.

But it is not only the lack of funds which hinders progress in infirmary nursing, another cause being the presence of untrained superintendents of trained nurses, and where this disastrous arrangement is in force it must always prove an obstacle to permanent improvements.

Unwieldy Proportions.

The size of workhouse infirmaries is seldom sufficiently considered in regard to the number of nurses needed. The blocks are often excellently designed, but how many times a day does the head nurse have to traverse the flights of stone steps that divide from each other the wards under her care?

Training in Infirmaries.

Where the matron is herself well trained, and has an adequate day and night staff of nurses, the workhouse infirmary furnishes an admirable school for probationers. There are, indeed, many such where the theoretical and practical teaching is in no way behind that of general hospitals. It is, therefore, probable that under the improved condition of things each year will see fresh recruits added to the noble army of workhouse infirmary nurses.

Help from Outside.

It is not only nurses that are needed to humanise asylums for the destitute and helpless, other influences are wanted to brighten monotonous lives. Visitors earnestly desiring to be helpful (not merely to while away an idle hour) are distinctly beneficial to the paupers, and so are suitable books and fresh flowers. Books made attractive by clear type and many illustrations should never be too heavy for feeble hands. Plenty of papers and journals are always acceptable, and might easily be secured, by a little forethought, at very small expense.

### Where to Go.

Tour for the Christmas Holidays.—The Polytechnic Committee are arranging a co-operative and educational tour to visit Rome, Naples, Florence, Venice, Pisa, Genoa, Milan, Italian Lakes, and Lucerne. All particulars can be obtained from the Secretary, 309, Regent Street, London.

Southport.—Grand New Infirmary Bazaar, in the Municipal Buildings, November 12th to 17th. Contributions of money and work are invited from all dwellers in the district

### Dress and Uniforms.

By a Matron and Superintendent of Nurses.

### I.—INTRODUCTORY.

Nothing strikes a casual visitor to London more forcibly than the large number of women which are to be seen walking about in the attractive and becoming garb of a nurse. No matter where one goes—to the park, the streets, or the play—always in evidence is the familiar bonnet with its white strings and flowing veil. The proposition which, not unnaturally, forces itself upon a reflective mind is this—either there are a large number of nurses out of employment or many persons are wearing a dress to which they have no sort of claim. There appears, unfortunately, to be only too strong grounds for assuming the latter of these two views to be the correct one. Numerous as the members of the nursing profession undoubtedly are, the numbers of the sick to be nursed are materially in excess of them. Indeed, the difficulty frequently arises of being able to command a sufficient supply of nurses to meet the demand. The writer well remembers, during the last outbreak of influenza, a lady coming to her one evening in despair, after having spent a long day in fruitless quest for a nurse, and offering fifty guineas if one could be spared her, in the emergency, as a valuable life depended on skilled attention. No doubt the majority of nurses pass through a slack time occasionally, but no useful and thoroughly efficient woman need be long without employment except by her own wish. For a solution of the problem we must seek another cause. The *Lancet* a short time ago drew attention to the fact that it was becoming fashionable for ladies to include in their wardrobe, among other articles of attire, the costume of a "hospital nurse."

A dress so neat and attractive could not for long hope to escape the flattery of imitation, discreditably as it reflects on those who thus wantonly lower the prestige of a noble profession by such thoughtless masquerading. There are women also, not of the best character, who assume the dress from unworthy motives, the object in their case being to render the wearer as conspicuous as possible, a consummation towards which the uniform only too readily lends itself. This, unfortunately, is one of the gravest scandals in connection with the matter, and points strongly to the advisability of patenting or registering some recognised uniform which could only be worn by professional nurses. It would be a very great comfort and protection to the bonâ-fide nurse if this could be done, and would be a means of effectually differentiating the "sheep from the goats" in the eyes of the public.

A large number of these pseudo nurses consist of domestic servants whose mistresses are chiefly to blame for the imposture, from an ignorant and misguided fancy that a distinctive uniform confers a dignity on an otherwise second-rate ménage. Advertisements for nursery-maids not unfrequently contain the stipulation that "one wearing nurse's uniform is preferred." All those who have the interests of the nursing profession at heart must deplore this tendency on the part of the public, and it behoves each one of us, by using the best means that are in our power, to educate them up to a sense of their responsibility in the matter. If it were considered as foolish and unprincipled for an unqualified person to wear nurse's uniform, as it would be for a civilian to parade in military attire, we should see and hear less about the number of nurses who at the present time appear to be at large.

It is argued that "sham nurses" can be detected by the manner in which they wear the uniform, but this is not always the case. Many genuine nurses, we regret to observe, are not so particular about their uniform as they might be, and they consequently give rise to false impressions and are liable to be branded as imposters. Indeed, it is not so very long ago that a trained nurse so far forgot her professional dignity as to appear before an audience, to whom she was lecturing, clad in a short black dress, somewhat *décolleté*, over which was arranged a large white muslin fichu, kept in position by a cameo brooch, while a caricature of a cap encircled a head which might easily have accommodated the proverbial four hens and a cock in the nursery rhyme. We therefore call on our professional sisters to exercise the greatest care in the selection of their uniform and to avoid all exaggeration in the shape of collars, bonnet strings, and cloaks. The habit of wearing aprons with the outdoor dress cannot be too strongly condemned. In the first place it renders them unpleasantly conspicuous, and in the second it is in the highest degree insanitary. What is to be thought of a trained nurse who in these days of antiseptic precautions deliberately conveys into the sick room the numberless micro-organisms and other impurities of the streets, or who may equally, on the other hand, carry from her patient the germs of disease to uninfected localities? The apron is necessarily a powerful medium for the conveyance of poisonous germs to or from the sick, and therefore should always be carefully laid on one side before going out. Uniform properly used is the greatest boon possible to a nurse. The few hours at a time which she occasionally has off duty do not permit of her making an elaborate toilette, the convenience therefore of a cloak and bonnet, which can be as quickly donned as it is doffed, may be imagined. Moreover, to nurses with limited means it is a very great consideration, as it always looks suitable, and never grows old-fashioned. The manner in which a nurse dresses her hair calls for a few special remarks. A neatly-arranged head is always a pleasant sight, and carries its own passport with it. Fashion should have no part in the coiffure of a nurse; the hair looks best coiled or plaited round the head, and drawn simply back under the cap or bonnet, as the case may be, in front. Touzzled fringes and unsightly chignons or "buns" simply vulgarise both the dress and the wearer. To some faces the hair cut short in front is undoubtedly an improvement, but the greatest discretion is necessary, and it is only admissible where the forehead is of such expanse that, to soften the face, the line between it and the cap may be broken by a neat arrangement of hair. In such a case tidiness is of the utmost importance, so that the refinement in bearing and appearance, after which nurses should so earnestly strive, may not in any way be impaired. Every detail of a nurse's dress should exhibit neatness, usefulness, and modesty. There is an infinite variety of shapes both in bonnets and cloaks, which we intend to deal with at length in subsequent articles. One of the best types of uniform consists, we think, in a neat close-fitting straw bonnet in either black or some other sober colour, tied with a small white cambric bow under the chin, and trimmed with velvet. Veils are by no means a necessary adjunct. As they are expensive, soon get shabby, and blow about most uncomfortably on a windy day—they may very well be discarded. A plain circular cloak always looks well and is besides independant of the vagaries of fashion. Voluminous cloaks, so largely affected by many nurses, are very unbecoming, as they give a bunchy, untidy look to the wearer. Especial attention should be given to the boots and gloves if a nurse wishes to establish a reputation for precision and forethought. Neat boots, with round, not pointed, toes, are the most suitable, they are likewise comfortable and in good taste. Black gloves always look well, and are more serviceable, if they are good ones, than any other.

(*Continued on page* vii.)

Dress and Uniforms—*Continued.*

### II.—INDOOR UNIFORMS.

In the first article we spoke of uniform in regard to its leading characteristics, and more especially the outdoor dress. We will now go a little more into detail, and consider the underclothing and indoor attire. Perhaps nothing appears to the observer as more extraordinary than the indifference with which a nurse wears a cotton dress all the year round without any harmful results. This, however, will not seem so strange when we consider the activity of the life. Regular exercise keeps the circulation in good order, and oftener than not when engaged in the more energetic discharge of her duties, as involved in ward work, a nurse complains of heat rather than of cold. She never has time to take a chill, for as soon as one thing is finished, something else claims her attention. A nurse who used to be a perfect martyr to chilblains while leading a life of ease at home, got rid of them entirely after she took up nursing. Some people, of course, require warmer clothing than others, they feel the cold more; but there is no reason why, because they wear a cotton dress, they should not be substantially clad beneath. Woven combinations, with high necks and long sleeves, are a very comfortable addition to the wardrobe in the winter time, and are not very expensive. Warm flannels and petticoats act as efficiently in keeping out the cold as a woollen dress, and are much more in accordance with hygienic principles. The prettiest and most serviceable materials for a nurse's dress are galatea, zephyr, or gingham. They wear longer than print, take less time to get up, and possess greater consistency. The dress may be divided into skirt and bodice; the latter should be plainly made, without any fulness, and buttoned down the front with either pearl or bone buttons. The bodice, which must fit without crease or wrinkle, is set into a narrow band at the waist, which also forms the band for the skirt. The sleeves demand very careful attention, or they are liable to uncomfortably restrain the use of the arm. Plenty of room, therefore, must be given in the armhole, and the sleeve shaped to fit it, so that there is no strain on the material when worn. The best shape is that known as the coat sleeve; it fits the arm somewhat closely, and is turned up at the wrist with a false hem. Though a slight easing of the sleeve on the shoulder is allowable as permitting greater freedom of movement, we must emphatically protest against anything approaching to fulness. Uniform owes one of its chief attractions to the fact that it is independent of fashion, and it should never be vulgarised by any weak attempt to follow it. A good deal of care is necessary with regard to the skirt. It should just clear the ground all round, it being turned up with a hem about three inches deep. A prudent nurse will do well to have her skirt made rather longer than she requires at first, and a tuck run loosely round, to make it the required length till it goes to the wash, when she can let down the tuck to allow for the shrinkage, which is almost inevitable. A nicely hung skirt contains, as a rule, one front breadth, gored into the waist, a side breadth, gored towards the back, the selvege side coming to the front; and either one and a half or two breadths at the back, according to the width of the material. The front and side pieces should be eased on to the band, into which the bodice has previously been fixed, and the other breadths must be gathered firmly and regularly on to it at the back. We would urge on nurses the very great advantage it is to them when they can make their own uniform, for it renders them independent as regards fashion, and also is a material saving to their pockets. So plain a dress requires little beyond neatness and exactness in cutting out and trying on, and affords besides a very pleasant and useful occupation for spare moments. To those who have never tried the experiment, but are anxious to begin, we should advise unpicking an old or cast-off bodice which fits well, and then pin it carefully into the material, cutting exactly by the pattern, which will also serve as a guide as to the amount of turning necessary. It can then be tacked together and is ready for fitting on. If these instructions are carried out minutely the operator cannot go very far wrong, even with the first attempt. Plenty of turning should be left in front which, when the bodice is tried on, must be pinned together so as to ensure the greatest accuracy in fitting. In the summer bodices can be made without lining, but in such a case must be very neatly finished off. For the winter we can recommend flannelette as a most comfortable and useful lining. Aprons are best made of white linen, and should come to within a couple of inches of the bottom of the dress. They look best slightly shaped to the figure in front, and gathered into the band at the back and sides. The bib should fit tightly across the chest, and on no account be too wide or it will bulge in a very ugly and ungraceful manner.

A Nurse's Apron.

When straps are worn they should not exceed two inches in width, and ought to cross in the middle of the back and fasten neatly on to buttons sewn about three inches on either side from the middle of the band behind. Large square pockets can be worn at the side, and are useful for many things. Plain collars and cuffs look better than anything in the fancy line, and should always be faultlessly clean. The squire collar is largely patronised and looks better than any other. Bands of linen about three inches wide are the most convenient kind of cuff, as they only need fastening on to a button attached to the sleeve.

### Presentation.

At Gravesend Hospital the matron and nurses recently presented the late House Surgeon (Mr. S. Woodhams), with a handsome microscope. Mr. Woodhams, in accepting the gift, said that he had spent five of the happiest years of his life in Gravesend, and acknowledged the unfailing kindness of the medical staff, and the pleasant hospitality which he had received from the residents of the town.

### A Novel Competition.

### DESIGN FOR A NURSES' HOME.

In The Hospital of July 15th we gave the opinion of Dr. Billings, the President of the International Congress at Chicago, that "it was about time that women manifested the latent power claimed from them, in a practical definite form. . . . He thought that congress was a very appropriate platform from which to invite the superintendents to elaborate plans and enumerate each item which a model nurses' home and training school should contain." The outcome of this discussion was the organization of "The Novel Competition for Nurses." The Editor of The Hospital offered two prizes of £15 and £5 respectively. The results of the competition have been interesting and fairly satisfactory, the second prize being awarded, after careful consideration, to Miss Hills. The judges were Dr. Henry M. Hard, Superintendent of the Johns Hopkins Hospital, Baltimore, and Miss Sophia S. Palmer, Superintendent of the Garfield Hospital, Washington. None of the plans sent in were considered worthy of a first prize, although they reflect great credit on the competitors, who have devoted so much care and patience to their neat drawings.

PLAN OF NURSES' HOME.—No. 1.

First Floor. Second Floor. Third and Fourth Floors.

Dotted lines show alterations that may be made if elevator is wanted.

A, Main vent flue. B, Vent flues. C, Smoke flues. D D, These two closets to be omitted if elevator is wanted.

### I.—By Mrs. E. J. Wakeman.

This plan shows a building in the form of a simple parallelogram 90 feet long by 40 feet wide, and with light on all four sides.

The building consists of four storeys, the lowest of which is, apparently, some two feet below the surface of the ground. Each of the upper floors is bisected from end to end by a wide corridor; on the lowest floor this corridor is divided unequally by the kitchen. The arrangement of the latter appears singularly ill-judged. There are no less than five doors, including one into the open area, which is apparently the tradesmen's entrance. Two of these doors, which face each other, are so placed that the space immediately in front of the range would certainly become a passage to and from the coal cellar. The water closets, five in number, none of which have direct light or ventilation, open into a "toilet-room," which opens directly on to the corridor. For overcoming the evil which must surely result from such a plan the author appears to rely on a ventilating shaft about one foot long, and formed within the thickness of a partition not more than six inches wide.

The second floor, which corresponds to what we should call the ground floor in England, contains a parlor, library, and lecture-room, sitting-rooms for superintendent nurse and matron, six bed-rooms, and two bath-rooms. Each of these latter contains also a w.c., and one of them opens directly out of the superintendent nurse's bed-room. The sitting-room for the latter official has its doors and windows so planned that it would be hopelessly uncomfortable, if not uninhabitable. Adjoining the second bath-room is a small closet, in which a slop sink is placed, absolutely in the dark.

The arrangements of the third and fourth floors are practically the same as on the first, except that the place of the sitting rooms and library-room is taken up by bed-rooms.

Altogether the plans provide accommodation for fifty nurses, each bed-room being intended to hold two beds, and the author states that as many storeys as need could be added, each story accommodating twenty nurses.

### II.—By Miss Hills.

This design is on an altogether different scale. The plan is in the form of an E. The entrance is in the centre of the upstroke, and the staircase forms the centre stroke. The front

(*Continued on page* ix).

door opens on to a hall of magnificent proportions, with the staircase immediately opposite. To the right are rooms for the directress and housekeeper, a store-room, and two smal l rooms for visitors. The right wing contains a large dining-room, with a china pantry adjoining, a back staircase, and the entrance from the garden. To the left of the entrance is a reading-room, with a lecture-room and general recreation-room adjoining. Two box-rooms and a lavatory and w.c. complete the accommodation in the left wing.

On the first floor are eighteen nurses' bed-rooms, nine sit-

PLAN OF NURSES' HOME.—No. 2.

Ground Floor Plan.

First Floor Plan.

Top Floor Plan.

ting-rooms for nurses, a bed-room and sitting-room for the directress, two "extra bed-rooms," four bath-rooms, linen-room, water-closets, sink-rooms, and back staircase.

On the top floor are ten nurses' bed-rooms, five sitting-rooms, one nurses' sick-room, an isolation-room, with bath-room and lavatory attached, and the kitchen offices and servants' bed-rooms. The bed-rooms in this plan are obviously intended for one nurse only, as they should be; but the provision of so many sitting-rooms, intended, apparently, to be used each by two nurses in common, is a luxury scarcely justifiable on grounds either of economy or necessity. While the scheme of the building, as a whole, is wasteful of space and extravagant, there are many features which are commendable, and evince much practical knowledge.

B No. 3.—A third plan is a variation of the last on much the same general outline. The kitchens here are placed on the ground floor instead of on the top floor, a plan which cannot be commended. With properly-constructed lifts there is no more expenditure of labour with an upper floor kitchen than with one below, and the gain in ventilation is immense. In this plan the entrance hall is of more modest dimensions, and there are two staircases instead of one. This latter plan has something to be said in its favour on the score of presenting greater facilities for escape in case of fire. The roofs of the two wings are in this plan utilised as gardens.

### A Visit to Edmonton Workhouse.

By a Wanderer.

While public opinion is being gradually aroused to the need for a better system of dealing with the worn-out veterans of industry, many of the boards of guardians throughout the country have been quietly and unostentatiously working out schemes of their own for the amelioration of the conditions of our aged and infirm poor. Among these, the Guardians of the Edmonton Union take a foremost place, and a visit to the workhouse and infirmary of the Union will well repay anyone. In everything that the Guardians have done they have been ably seconded by the Matron.

On arriving at the Union I was taken by the Matron to the female side of the infirmary. Owing to the extensive alterations which were going on, the first impression was certainly one of discomfort, but on entering a ward this idea was banished. The long room was bright, airy, and cheerful, and the old faces framed in their white frilled nightcaps looked wonderfully contented. One or two were reading, and some were passing the time with sewing.

Some of the wards were small, only containing six or seven beds, but their appearance also was bright and airy, the walls being painted in artistic colours, not white-washed, as one expected. The bath and lavatory accommodation was superior to that of some of our city hospitals, and the Matron said that each patient had a bath at least once a fortnight, and adequate changes of linen. The Matron is assisted by six trained nurses, who have a considerable amount of pauper help. One of the trained nurses is always on duty at night.

The dormitories in the workhouse itself differ very little from the wards. The beds are springs, each with a pair of sheets, a pair of blankets, and a warm quilt. Lying on each bed there was a red-frilled nightgown-bag, with the number of the bed worked on it, and a similar bag was hanging at each bedside containing two combs and a brush. Each person is provided with a clean towel once a week, and the bath accommodation is very good.

The new dining-hall, which is nearly completed, looked huge yet not too large for the dining-tables of the inmates, the number of the latter accommodated in the house being 750.

Besides the dining-hall there is a new kitchen, scullery, and two laundries, one for the officers and the other—a much larger one—for the inmates, each containing a large drying press. As the present laundry accommodation was inadequate, this part of the alterations was urgently necessary.

Leaving the main building for the nursery, the first feeling was one of disappointment. The children were having dinner, at a desk close gainst the wall. But the cheerlessness was striking. The building was a corrugated iron one, standing apart, and originally intended as a ward for infectious cases. It certainly was not an ideal nursery, and even more dull was the room in which the infants dwelt; the long cradle, which held four or five little mites, rocked up and down, not from side to side, was very suggestive of sea-sickness. Close by, a second wooden cradle held twins, whilst another baby, seated on the floor, appeared to be acting as nurse, and the little creature managed her charges well.

Considering the superior arrangements and the well-ordered aspect of the other parts of the house, the nurseries at Edmonton are certainly disappointing. Yet where so much has been already done to ameliorate the condition of the inmates, it is safe to prophecy that at no distant date more cheerful surroundings will be provided for the little children who are paupers through no fault nor willingness of their own.

### Trained Nurses as Stewardesses.

This favourite old scheme of The Hospital seems to have come into fresh favour recently, a number of questions on the subject finding their way into the Editor's letter box. Good nurses may make good stewardesses, but unmethodical or indolent ones are certain to fail under that test of stern discipline and keen scrutiny to which the work is subjected on board ship. There is no one else to share the blame of faults of omission or commission. Duty is far too accurately defined to leave any loopholes. But relaxation and meals are also punctual, and the life of a trained stewardess may be made as pleasant as it is healthful. For information as to pay and regulations, application should be made at the offices of any of the large shipping companies.

### Male Nurses.

England is still behind America and Germany in the matter of educating male nurses. In both those countries men can secure a training in general nursing, which is here withheld from them. A correspondent recently reminded us that soldiers, under certain conditions, obtained an efficient training in all departments of hospital nursing, but this is of little value to civilians desirous of taking up nursing minus soldiering! It certainly seems hard that systematic instruction should be withheld from candidates who might be made by it into useful attendants for many male patients.

### Appointments.

Forfar Infirmary.—Miss Foulkes has been appointed Matron of this infirmary. She was trained at the Derbyshire Royal Infirmary, had charge of wards there, and took the duties of Night Superintendent on various occasions. Miss Foulkes joined the Scottish branch of the Queen Victoria Jubilee Institute at Edinburgh, and after six months' work there she was drafted to Forfar, where she has done two years' district nursing. Thorough knowledge of housekeeping and general management are invaluable additions to Miss Foulkes' nursing experience, and the Forfar Committee may be congratulated on the matron they have secured for their infirmary.

Kingston Hospital, New York, U.S.A.—Miss Sarah Henry has been recently appointed Matron of this hospital. She was trained at the Royal Infirmary, Liverpool, and was Ward Sister there for several years. She also had experience in district and private nursing in connection with the same institution. Miss Henry was afterwards Night Superintendent of the Western Infirmary, Glasgow. We congratulate her on her appointment, and wish her success in her new work.

Islington Workhouse Infirmary.—Miss Emily Jones has been appointed matron of this infirmary. She was trained at St. Bartholomew's Hospital, and subsequently became night superintendent at Chelsea Infirmary, from whence she went as assistant matron to Homerton. We wish Miss Jones every success, and congratulate the institution which has secured her services.

Livingstone Cottage Hospital, Dartford.—Miss Mary Pavyer has been appointed matron of this new hospital. She was trained at Tottenham Hospital where she remained for nearly four years, and was afterwards nurse at Westminster Hospital for over three years. For the past three months Miss Pavyer has been temporary nurse-matron at the Bexley Cottage Hospital. Miss Pavyer takes with her many good wishes for her continued success.

Infectious Diseases Hospital, Bannockburn. — Miss Agnes Dempster, of the Kilmarnock Fever Hospital, has been appointed Nurse-Matron at Bannockburn. We congratulate her on her appointment.

### Wigmore Nurses' Co-operative Institution.

Two years ago the Wigmore Nurses' Institution changed hands, and commenced work on the co-operative principle. The nurses, forty-eight in number, take their own earnings, and pay half a crown in the pound to the Lady Superintendent, who manages the comfortable homely looking house at 2, Bulstrode Street, Welbeck Street. Board and lodging at a fixed rate are provided for the workers between their cases. The scale of fees paid by patients varies according to the illness, ordinary medical cases being £1 11s. 6d. to £2 2s., and mental and infectious ones £2 2s. to £3 3s. per week. The results under the present system are considered satisfactory, the average fees received by the nurses may be taken as two guineas a week, and the time when the nurses are without work is said to be only such periods as form the reasonable holidays which every private nurse should allow herself. The old-fashioned house in Bulstrode Street has been made the best of, and the nurses' cubicles are very airy and clean, the sitting and dining rooms being on the ground floor. The Superintendent is able sometimes to supply nurses for permanent cases at fixed salaries.

### Novelties for Nurses.

The delightful fabrics supplied by Messrs. Egerton Burnett, Wellington, Somerset cannot fail to commend themselves to nurses. Excellent washing materials for uniform dresses are supplied at most reasonable prices. There are stripes of various widths and in all shades of blue and grey, whilst for those who prefer plain colours an equally good selection is provided. The serges made by this well-known firm are proverbially sound and serviceable, and some of the patterns sent out by them this autumn are irresistibly attractive. They vary considerably in texture and price, so that all purchasers are sure of finding something to suit them. Uniforms, gowns and cloaks and holiday costumes can be promptly selected, for everyone knows that few costumes are more becoming than those made of good serge. Washable tweeds are also supplied by Messrs. Egerton Burnett, especially designed for nurses' use, and waterproof materials for light-weight sanitary cloaks are deservedly popular, made up into any shape desired at moderate cost.

### Massage.

Certain disclosures brought before the public recently cannot fail to have a deterrent effect on the adoption of massage as a means of livelihood. Doubtless masseurs and masseuses of good repute have suffered little by the unpleasant revelations, but novices may well take warning and secure reliable advice before entering into engagements or commencing a course of training. Massage is made part of the training at many general hospitals now, and a thorough knowledge of the art as practised under medical supervision is valuable to nurses.

### Wants and Workers.

The Lady Superintendent of the Coleraine Cottage Hospital, co. Derry, would gratefully accept six empty "natural seltzer water" bottles, to be used as foot-warmers by the patients.

Can anyone give information as to a home for a girl of seven, who is not deaf but almost dumb? Small weekly payment can be made.— F. M. D.

For Everybody's Opinion, see p. xi.

### Everybody's Opinion.

[Correspondence on all subjects is invited, but we cannot in any way be responsible for the opinions expressed by our correspondents. No communications can be entertained if the name and address of the correspondent is not given, or unless one side of the paper only be written on.]

### NURSES IN PARIS.

"An Outsider" writes: I wish to publicly thank the "Levick Nurse" for her letter in your last issue. Although I am only an outsider, as a resident in Paris I have heard from time to time pitiful stories from nurses who have been obliged to leave the institution without the money which was due to them. Realising the position of an English nurse in a foreign land, surely it is time that steps were taken to prevent unscrupulous persons from getting nurses to Paris under false pretences. In again thanking her for her letter, I can say your correspondent has dealt too leniently with her subject. To be sent (through a charitable fund, and by no fault of her own) back to England whilst £12 14s. was owing to her, was indeed a galling position to be placed in.

### STAMPED AND ADDRESSED ENVELOPES.

"A Constant Reader," writes:—Complaints are not infrequently heard from nurses seeking work that when they answer advertisements enclosing stamped envelope for reply, the courtesy of that reply is not forthcoming. Nurses, however, are not the only victims in this respect! Needing lately to increase my staff of private nurses, I myself answered various advertisements, duly enclosing stamped envelope, but although this has been annexed by the nurse in several cases, no reply of any kind has been vouchsafed to me. I should be glad to know if other readers of The Hospital have had similar experiences, and also if it is not usually considered mere ordinary civility to send at least a few words of reply when an addressed and stamped envelope is enclosed?

### NURSES' HOLIDAYS.

Miss Dunwoodie writes: I have just returned from a holiday spent partly in Liverpool, and I think the kindness of the Liverpool Steamship Company should be acknowledged. By applying at the offices nurses can have tickets at half fare to any of the seaside places along the Menai Straits, and the "two hours' Channel cruise" is a free trip to nurses. My sister, who has an invalid and nurses' home, had two free tickets sent for the trips to Menai Bridge, and with thoughtful kindness they were undated. Messrs. Hengler sent us two tickets for their circus. Other people may show similar kindnesses, but of these I speak from personal experience and with grateful acknowledgment. What a boon to tired nurses to have such delightful airings on the water without any drain on a slender purse.

### A NURSE'S HOLIDAY.

Sister Dora writes: I should like to tell your readers a little about the very pleasant month's holiday I have just spent at the Convalescent Home for Women and Children, New Brighton, Cheshire. As there has been a new wing opened lately for first class patients, I decided to go there as I was feeling tired and overworked, and wanted to go somewhere where I could be "looked after" a little. I arrived at New Brighton in the afternoon, and soon reached the home, which is only about two minutes' walk from the station, where I was very kindly welcomed by the Matron, and taken to my snug little well-furnished room on the second corridor. There were about twenty-two first class patients besides myself, and I soon found that we all met for meals in a large and comfortable dining-room. There is a drawing-room for our use, with sofas, easy chairs, piano, &c., also a delightful little boudoir where one can retire to write letters or read. In addition to this, there is a splendid large room, called the recreation hall, where all who like can go in the evenings or on wet days to dance, sing, or play games, &c. Those who need it can have medical advice and careful nursing but nearly all the patients, like myself, seemed to be just wanting a complete change in every sense of the word, and after a few days quiet were quite ready to join in all the fun and amusement that was continually going on. As the home is well warmed throughout with the hot air apparatus, it makes it a very comfortable residence in the winter months as well as the summer. I could tell much more about the delightful sea trips and donkey rides on the sands, the sea bathing, &c., if space permitted, but I cannot conclude without telling my readers how very kind and considerate I found the matron and her assistants, and what a real "home" they contrive to make it. Anyone can be admitted by writing to the Matron and filling in the form of application, and as the terms are so moderate (one guinea per week) many, perhaps, will be glad to avail themselves of a home so well suited for tired nurses.

### ON GENERAL NURSING.

"Nurse L." writes: I should be glad for Dr. Rowland Humphreys to receive my thanks, and those of other nurses, for the very interesting and instructive papers on "General Nursing" written by him for The Hospital. They cannot fail to have helped many nurses, and the article on peritonitis strikes me as an especially valuable one.

### Notes and Queries.

### Queries.

(1) *India.*—Can you tell me where to apply for information as to getting an appointment on the Indian Nursing Staff? I have been advised to ask you about it.—*H. E. T.*

(2) *France.*—I shall be glad to know of an institution in France which sends out English private nurses.—*Traveller.*

(3) *Midwifery.*—I have a L.O.S. diploma, and wish for a year or two's general training in a workhouse infirmary. Where should I be likely to get it in London?—*Nurse Jane.*

(4) *Nice.*—Information wanted about the Hollond Institute.—*Nurse Elsie.*

(5) *Stewardess.*—How can a trained nurse become a stewardess?—*L. E. T.*

(6) *Nurses' Library.*—Can you tell me how I can see the books most suitable for a nurses' library? Catalogues are useful, but I should prefer to see the volumes before purchasing.—*Provincial Superintendent.*

(7) *Matrons' Council.*—What is the fee for admission to this, and who is eligible?—*Aspirant.*

(8) *Medical Nursing.*—When will the "Notes on the late Dr. James Anderson's Lectures to Nurses" be published? I have only seen a preliminary notice of them as yet.—*One who has Nursed for Him.*

(9) *Leaflets.*—Where can leaflets be obtained containing hints on nursing, disinfection, and kindred subjects for distribution amongst the poor?—*M. R. S.*

(10) *Nursing.*—Kindly inform me if Miss Hampton's book, "Nursing," can be procured in England?—*U. S. A.*

### Answers.

(1) *India (H. E. T.).*—If you have had three years' training in a good general hospital, you should write to the Director-General of the Army Medical Department for a form of application. The Up-country Nursing Association has been recently formed for Europeans in India, and particulars can be obtained from the Hon. Treasurer, the Hon. Mrs. Neville Lyttelton, 21, Carlton House Terrrace, or Major-General J. Bonus, R.E., The Cedars, Strawberry Hill (Hon Secretary).

(2) *France (Traveller).*—If you write to the Directress of the Hollond Institute at Nice you will find out the various localities in which branches have been established, and also learn whether there are any vacancies this year. See our advertisement columns.

(3) *Midwifery (Nurse Jane).*—Apply to the Matron of the Holborn Union Workhouse Infirmary, or any other where you are sure of the Matron being herself a fully-trained nurse.

(4) *Nice (Nurse Elsie).*—See answer 2.

(5) *Stewardess (L. E. T.).*—Apply at the offices of the large shipping companies, of which you will find the names in most daily papers. You must remember that the duties of a stewardess are as real as her privileges. She has to make many beds, which requires a great deal of knack when they are berths, and she has to be punctual, precise, and obedient. Her meals and her times off duty are seldom interfered with, and the life is a particularly healthy one for a practical working nurse who is a good sailor.

(6) *Nurses' Libraries (Provincial Superintendent).*—Why do you not call at the offices of the Scientific Press, 428, Strand, and see their unique collection of suitable books?

(7) *Matrons' Council (Aspirant).*—We believe the fee to be 5s. member's annual subscription, and 2s. 6d. for associates. We are told that any trained "sister" or "staff nurse" is eligible, therefore the title is rather wide of the mark.

(8) *Medical Nursing (One who has Nursed for Him).*—They are now ready. You had better write to the publisher, H. K. Lewis, 136, Gower Street, W.C.

(9) *Leaflets (M. R. S.)*—The Ladies' Sanitary Association, 22, Berners Street, publishes numerous health tracts, and these and other pamphlets can be supplied, if desired, through the Scientific Press.

(10) *Nursing (U. S. A.)*—A second edition of Miss Hampton's book has just been brought out by the Scientific Press.

## Figures and Tables

**Figure f1:**
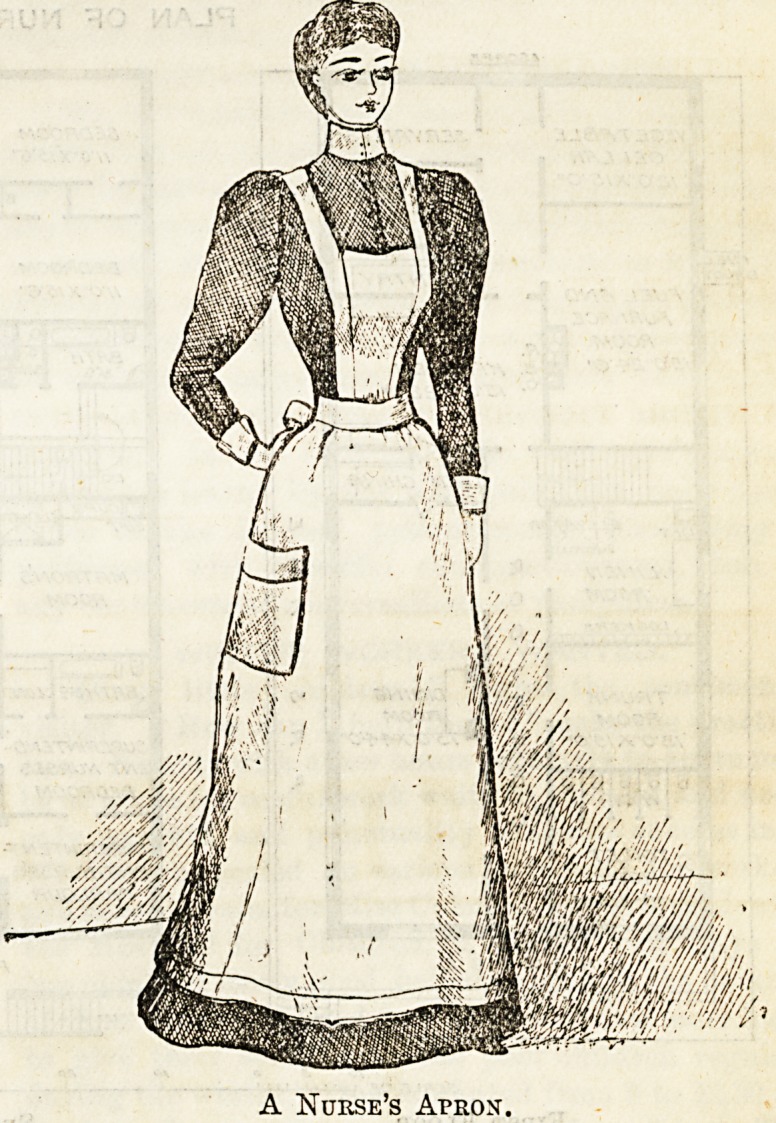


**Figure f2:**
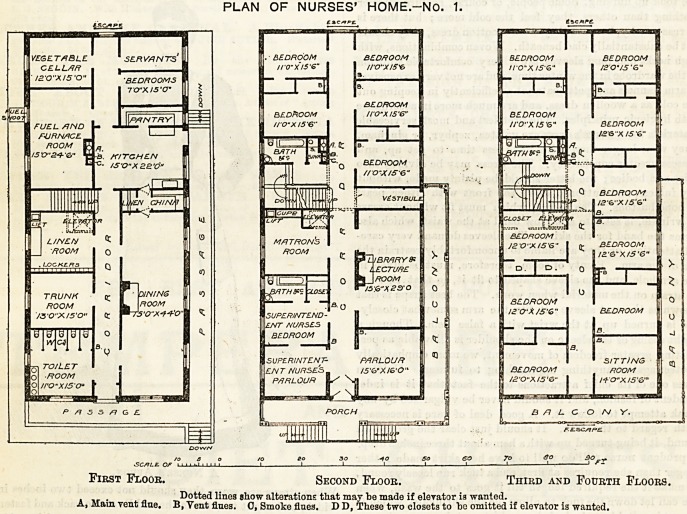


**Figure f3:**